# Naturally occurring cell adhesion inhibitors

**DOI:** 10.1007/s11418-018-1220-z

**Published:** 2018-05-19

**Authors:** Satoshi Takamatsu

**Affiliations:** Division of Natural Medicine and Therapeutics, Department of Clinical Pharmacy, School of Pharmacy, Showa University, 1-5-8 Hatanodai, Shinagawa-ku, Tokyo, 142-8555 Japan

**Keywords:** Cell adhesion, Inhibitor, Natural products, LFA-1, ICAM-1, HUVEC, PMA, LPS, TNF-α

## Abstract

This paper reviews naturally occurring cell adhesion inhibitors derived from a plant, microbial and marine origin. Plant-derived inhibitors are classified according to a type of structure. Microbially and marine-derived inhibitors were described according to age. In addition, effects of inhibitors on cell proliferation and that of standards on cell adhesion are listed as much as possible.

## Introduction

Cell adhesion molecules (CAMs) such as intercellular adhesion molecule 1 (ICAM-1, CD54 and immunoglobulin), vascular cell adhesion molecule 1 (VCAM-1) and E-selectin (CD62E) are critical in the regulation of immune response and inflammation. The extracellular interactions between specific CAMs expressing on the endothelium and leukocytes mediate leukocyte entry into tissues, T cell proliferation, and antigen presentation [1–4]. The key event in autoimmune disease is the migration of leukocytes to the disease site. Agents that inhibit leukocyte adhesion, transmigration and expression of related CAMs represent therapeutic potential as immunosuppressives and anti-inflammatory drugs. The major adhesive force for lymphocyte extravasation from the blood stream into tissue site is the protein–protein interaction of the adhesion molecules lymphocyte function-associated molecule 1 (LFA-1, CD11a/CD18 and β2 integrin) and its endothelial counter-receptor ICAM-1 [5, 6]. Monoclonal antibodies to ICAM-1 have been shown to inhibit lymphocyte transendothelial migration and have yielded very promising results in clinical trials for rheumatoid arthritis and organ transplantation [7, 8]. Therefore, the search for specific inhibitors of integrin-mediated cell adhesion with a small molecule in expectation of anti-inflammatory and anti-metastatic drugs started in the 1990s.

Generally, a cell adhesion inhibitor is categorized as target for cell–cell adhesion and for expression of cell adhesion molecules. Though certain small molecules such as flavonoids [9, 10] and others [11, 12] affecting expression of cell adhesion molecules are known, specific inhibitors for cell–cell contact are limited here in the review, as possible.

So far, some cell adhesion inhibitors based on synthetic methods and computer-aided drug design have been developed [13, 14]; however, natural products can still make unexpected structural discovery possible and they are believed to be a reservoir of resources for new types of drugs. This review introduces cell adhesion inhibitors focusing on those of naturally occurring plant, microbial and marine origin.

## Cell adhesion inhibitors of plant origin

### Terpenoid-sesquiterpene

*Chloranthus japonicas* Sieb. (Chloranthaceae) is a perennial herb that grows in the southern part of Korea, Japan and China. It has been used for boils, dermatological disorder, and enteric fever in Korea as a folk remedy. Three active dimeric sesquiterpenoids of shizukaol B (**1**), cycloshizukaol A (**2**) and shizukaol F (**3**) were isolated from the MeOH extract of roots of *C. japonicus* [15]. These compounds inhibited phorbol 12-myristate-13-acetate (PMA)-induced homotypic aggregation of human promyelocytic leukemia (HL-60) cells without cytotoxicity with MIC values of 34.1 nM (**1**), 0.9 µM (**2**) and 27.3 nM (**3**), respectively. Although **1**–**3** did not affect the direct binding of LFA-1 to ICAM-1, these compounds markedly inhibited ICAM-1 expression in HL-60 cells in a dose-dependent manner. On the other hand, when human umbilical vein endothelial cells (HUVECs) were pretreated with **1**–**3** and stimulated with tumor necrosis factor α (TNF-α), adhesion of THP-1 cells to HUVECs decreased in a dose-dependent manner with IC_50_ values of 54.6 nM, 1.2 µM and 34.1 nM, respectively. In fact, **1** inhibited TNF-α-induced surface expression of the ICAM-1, VCAM-1 and E-selectin in HUVECs with IC_50_ values of 5.4 nM, 13.6 µM and 95.6 nM, respectively.

α-Iso-cubebene (**4**), a novel cubebene sesquiterpene from *Schisandra chinensis* (Schisandraceae), attenuated the activities of adhesion molecules in TNF-α-stimulated HUVECs [16]. α-Iso-cubebene (**4**) significantly suppressed the TNF-α-induced cell surface expression of VCAM-1 and E-selectin (43.8 and 29.6% inhibition, respectively) at 25 μg/mL, but not ICAM-1 expression. α-Iso-cubebene (**4**) attenuates TNF-α-stimulated endothelial adhesion to monocytes by inhibiting intracellular reactive oxygen species (ROS) production, the activation of redox-sensitive nuclear factor κB (NF-κB) transcription factor and expression of VCAM-1 and E-selectin.


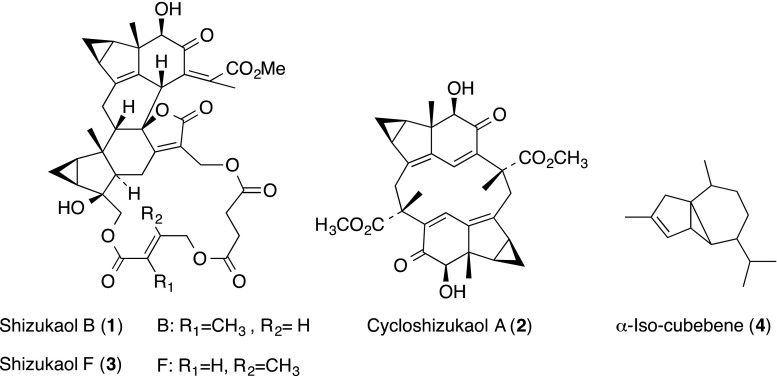


### Terpenoid-diterpene

Four clerodane diterpenes, 18,19-diacetoxyclerodane 18,19-oxide acetals, casearinols A (**5**) and B (**6**), and casearinones A (**7**) and B (**8**), were isolated from the leaves of *Casearia guianensis* (Flacourtiaceae) [17]. Compounds **5**–**8** inhibited the binding of LFA-1 to ICAM-1. Quantitative data were obtained for casearinol A (**5**), which inhibited the binding of LFA-1 to ICAM-1 in a dose-responsive manner, yielding an IC_50_ of 50 µM. This is the first report of immunomodulatory activity for this class of diterpenes.

Andrographolide (**9**), an *ent*-labdane diterpenoid lactone isolated from the Chinese official herbal *Andrographis paniculata* (Acanthaceae), has been reported to have anticancer activity [18–20]. Jiang and co-workers reported that **9** inhibited the adhesion of gastric cancer cells with a highly expressing level of sialyl Lewis^X^ (SLe^X^) to the TNF-α-stimulated human endothelial cells by blocking E-selectin expression in a dose-dependent manner, in a concentration range of 1–10 µM [21].


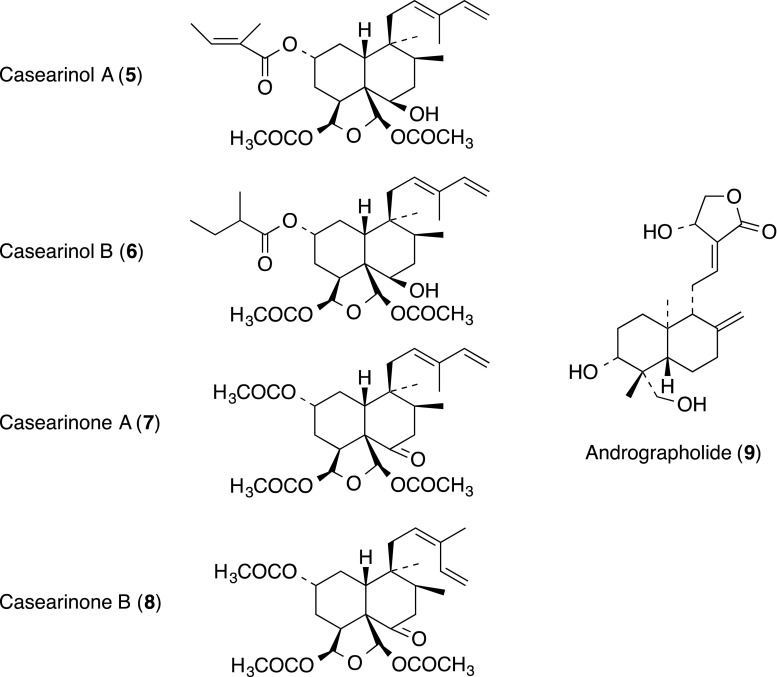


### Terpenoid-triterpene, steroid and related compound

Cucurbitacin E (**10**) was isolated from CH_2_C1_2_ extract of the stem and leaves of *Conobea scoparioides* Benth. (Scrophulariaceae) as an antagonist of CD18-mediated cell adhesion. Cucurbitacin E (**10**) is a tetracyclic triterpenoid with an unsaturated side chain present in various plant families such as the Cucurbitaceae, Scrophulariaceae, Euphorbiaceae, Liliaceae and Elaeocarpaceae. Cucurbitacin E (**10**) and five related analogues, cucurbitacins B (**11**), I (**12**), D (**13**), L (**14**) and R (**15**) obtained separately, were tested in the cell adhesion assay. Compounds **10**–**13** showed inhibition of JY/HeLa cell binding through LFA-1/ICAM-1-mediated adhesion, with IC_50_ values of 0.18, 0.30, 0.95 and 1.36 µM, respectively. Cucurbitacin E (**10**) was demonstrated to inhibit cell adhesion to HeLa cells by interfering with LFA-1 and not ICAM-1 [22].

Touihri-Barakati and co-workers reported that cucurbitacin B (**10**) from the leaves of Tunisian *Ecballium elaterium* (Cucurbitaceae) showed anti-integrin activity on human glioblastoma U87 cells, without being cytotoxic at concentrations up to 500 nM [23].


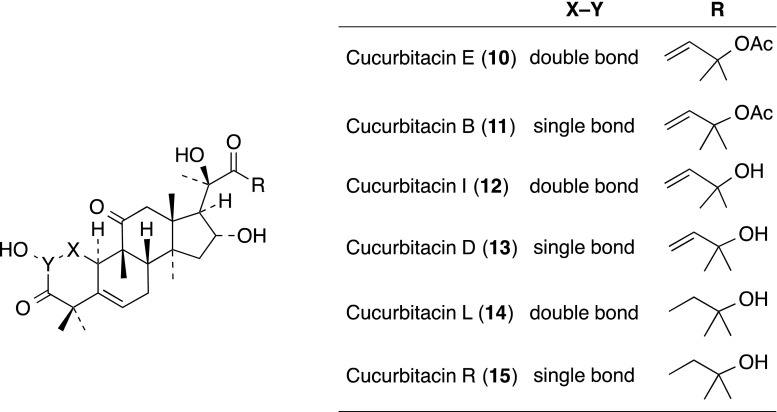


The extract from the root of *Trichilia rubra* (Meliaceae) was identified as having potent inhibitory activity in a bioassay for LFA-l/ICAM-I-mediated adhesion of JY and HeLa cells [24]. A series of *seco*-limonoids (**16**–**22**) with uncommon hemi *ortho* ester A-rings, was isolated. Compounds **16**–**22** exhibited potent inhibitory activity in the LFA-l/ICAM-1-mediated cell adhesion assay with IC_50_ values in the range of 10–25 nM. None of the compounds showed cytotoxicity at concentrations up to 20 µM.

The tetracyclic triterpene euphol (**23**) is the main constituent found in the sap of *Euphorbia tirucalli* (Euphorbiaceae), widely known in Brazilian traditional medicine for its use in the treatment of several kinds of cancer. The effect of euphol (**23**) on experimental models of colitis and the underlying mechanisms involved in its action has been reported [25]. The euphol (**23**) decreased lipopolysaccharide (LPS)-induced monocyte chemotactic protein 1 (MCP-1), TNF-α, interleukin 6 (IL-6) and interferon γ (IFN-γ), but increased IL-10 secretion from bone marrow-derived macrophages in vitro, and markedly inhibited both selectin (P- and E-selectin) and integrin (ICAM-1, VCAM-1 and LFA-1) expression in colonic tissue. Moreover, euphol (**23**) treatment markedly inhibited the activation of NF-κB in mouse colon tissue.

α-Tomatine (**24**), a glycoalkaloid isolated from *Lycopersicon esculentum* Linn, was reported to inhibit the PMA-induced abilities of adhesion, morphology/actin cytoskeleton arrangement, invasion and migration by cell–matrix adhesion assay, through blocking protein kinase Cα (PKC-α), extracellular signal-regulated kinase (ERK) and NF-κB activation. [26]


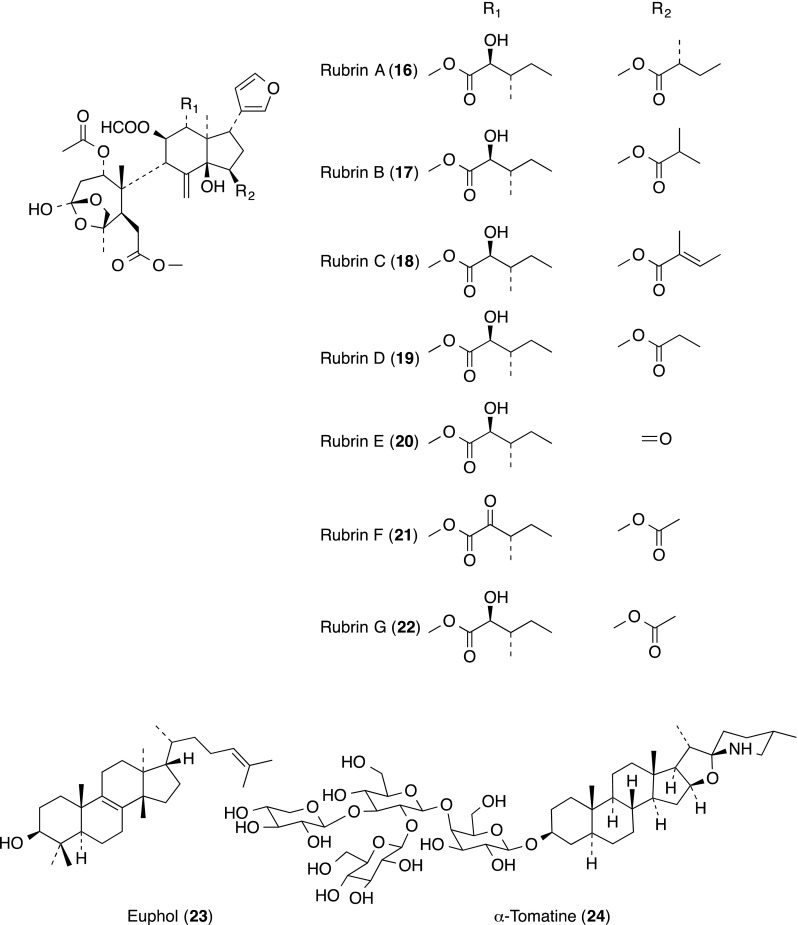


### Lignane

Manassantin A (**25**) and B (**26**), dineolignans isolated from *Saururus chinensis*, inhibited PMA-induced ICAM-1/LFA-1-mediated homotypic aggregation of HL-60 cells without cytotoxicity and with MIC values of 1.0 and 5.5 nM, respectively. After pretreating HUVECs with **25** and **26** followed by stimulation with TNF-α, adhesion of human acute monocytic leukemia cell line THP-1 to HUVECs decreased in a dose-dependent manner with IC_50_ values of 5 and 7 ng/mL, respectively, without cytotoxicity [27]. Both **25** and **26** also inhibited TNF-α-induced up-regulation of ICAM-1, VCAM-1 and E-selectin.


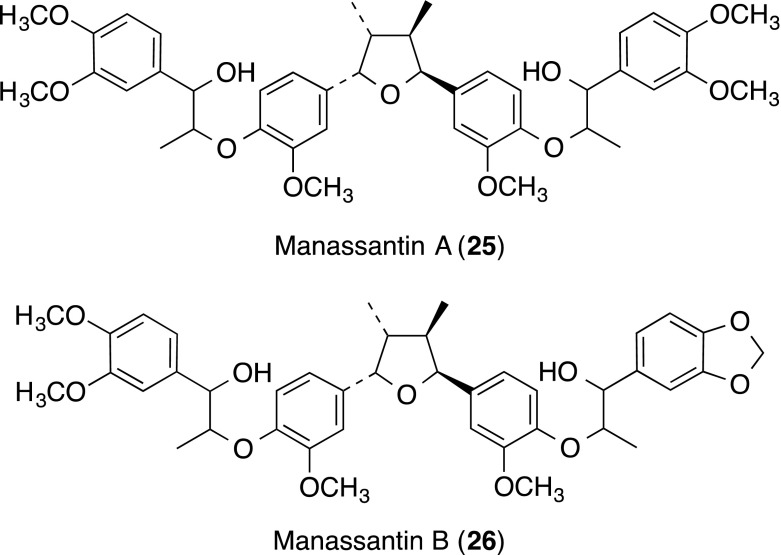


### Flavonoide

Astilbin [3,3′,4′,5,7-pentahydroxyflavanone 3-(6-deoxy-(l-mannopyranoside)] (**27**) from the rhizome of *Smilax glabra* (Liliaceae) was demonstrated to show a selective immunosuppressive feature [28]. The effect of **27** on concanavalin A (Con A)-induced liver injury by focusing on the TNF-α production and T lymphocyte adhesion was investigated. Inhibitory effect of **27** on the adhesion of Con A-activated human Jurkat T cells to endothelial cell line ECV-304 was reported.


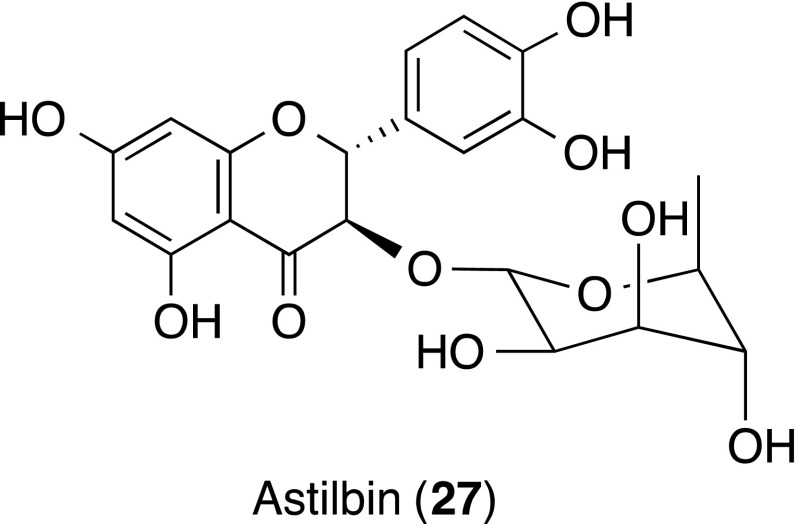


### Alkaloid

Piperine (**28**) and ethyl 3′,4′,5′-trimethoxycinnamate (**34**; the structure is shown in a section of other compounds described below) from the combined hexane and chloroform extracts of *Piper longum* (Piperaceae) were isolated as potent inhibitors of cell adhesion molecules on HUVECs [29]. Both **28** and **34** inhibited the TNF-α-induced expression of ICAM-1 at IC_50_ values of 45 and 25 µg/mL, respectively. In further study, **34** significantly blocked the adhesion of neutrophils to endothelium in a concentration-dependent manner. Compound **34** also significantly inhibited TNF-α-induced expression of VCAM-1 and E-selectin at 50 µg/mL. To elucidate its structure–activity relationship, effects of synthesized analogues of **34** and its thio, thiono analogues, and synthesized 7-hydroxy-4-methylcoumarin derivatives on cell adhesion molecules were studied [30, 31].

Lee and co-workers reported four quinolone alkaloids (**29**–**32**) isolated from the methanol extracts of Evodiae fructus, as the specific inhibitor on the binding of LFA-1 and ICAM-1 [32]. Evodiae fructus is natural medicine originated from *Evodia rutaecarpa* (Juss.) Benth. (Rutaceae), which has been used for treatment of gastrointestinal disorders and headaches, as an analgesic and antiemetic, and for amenorrhea in Korea.

The four quinolone alkaloids inhibited the interaction of sICAM-1 and LFA-1 in THP-1 cells at IC_50_ values of > 150 (**29**), 109.8 (**30**), > 150 (**31**) and 40.9 μM (**32**), respectively [vs. lovastatin (**66**) as a positive control, IC_50_ 33 µM]. On the other hand, they had no effect on direct binding assay using sVCAM-1 and E-selectin. They did not show cytotoxicity at the concentrations employed in the study (ca. 70–80% of THP-1 cell viability at 150 µM).

Among four quinolone alkaloids (**29**–**32**), cell adhesion inhibitory activity was suggested to be positively influenced by the presence of a double bond and an increase in aliphatic side chain length.

Castanospermine (**33**) is an indolizidine alkaloid originally isolated from the seeds of *Castanospermum austral* (the Australian Moreton Bay Chestnut, Fabaceae). Effects of **33** in a range of concentrations from 16,384 to 0.25 μM on mononuclear/endothelial cell binding and expression of their cell adhesion molecules were reported [33]. Upon HUVECs, **33** reduced expression of E-selectin, ICAM-1, ICAM-2 and platelet endothelial cell adhesion molecule (PECAM)-1, but increased it for P-selectin. Upon peripheral blood mononuclear cells, **33** reduced expression of L-selectin, LFA-1α, very-late antigen 4 (VLA-4; integrin α4β1), macrophage adhesion ligand 1 (Mac-1) and complement receptor 4 (CR-4; CD11c/CD18), but increased expression of P-selectin glycoprotein ligand 1 (PSGL-1) and PECAM-1. Similar changes of expression were found in the subset of lymphocytes and monocytes, but the reductions in LFA-1α and VLA-4 on lymphocytes and Mac-1 (CD11b/CD18) and CR-4 on monocytes were greater.


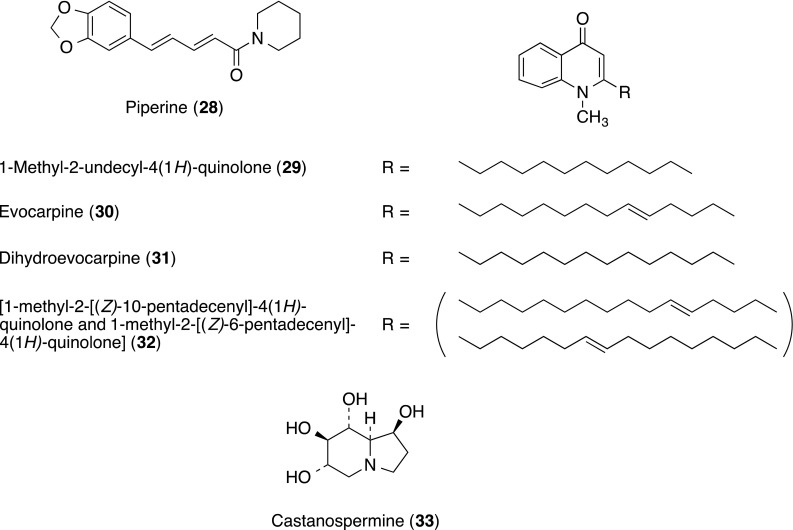


### Other compounds

Lee and co-workers found an inhibitory effect of methanol extract of *Rheum undulatum* (Polygonaceae) rhizomes on cell adhesion in search for anti-inflammatory or anti-metastasis agents, and isolated six stilbenes from the by bioactivity-guided fractionation. Six stilbenes were identified as desoxyrhapontigenin (**35**), rhapontigenin (**36**), *trans*-resveratrol (**37**), piceatannol (**38**), piceatannol-3′-*O*-β-D-glucopyranoside and isorhapontin. Among them, **35**–**38** inhibited the direct binding between sICAM-1 and LFA-1 of the THP-1 cells in a dose-dependent manner with IC_50_ values of 50.1, 25.4, 33.4 and 45.9 μM, respectively (Table 1). Compounds **36**, **37** and **38** also had an inhibitory effect on direct binding between sVCAM-1 and VLA-4 of THP-1 cells [34].

**Table 1:** Inhibitory effect of **35**–**38** on cell adhesion-mediated LFA-1/sICAM-1 and E-selectin/VCAM-1

	LFA-1/sICAM-1 (µM)	E-selectin/sVCAM-1 (µM)
**35** ^a^	50.1	Inactive
**36**	25.4	> 100
**37**	33.4	> 100
**38**	45.9	44.1
Lovastatin (**66**)	57.2	–

^a^Compound **35** showed cytotoxicity at 100 µM

In addition, compound **38** can interfere with the binding between integrin (LFA-1 and VLA-4) and immunoglobulins (ICAM-1 and VCAM-1). A lovastatin (**66**) was used as a positive control for the binding between sICAM-1 and LFA-1 of the THP-1 cells (IC_50_ 57.2 µM; Table 1).

Sparstolonin B (**39**) is an isocoumarin compound isolated from the tubers of both *Sparganium stoloniferum* and *Scirpus yagara*. Sparstolonin B (**39**) inhibited LPS-induced expression of ICAM-1 and VCAM-1 in HUVECs at 10 and 100 µM, respectively [35].

Sparstolonin B (**39**) significantly suppressed the adhesion of THP-1 cells to LPS-activated HUVECs at a concentration of 100 µM. The inhibitory effect of **39** on LPS-induced phosphorylation of extracellular signal-regulated kinase (Erk1/2) and serine/threonine kinase (Akt, protein kinase B) was also reported.


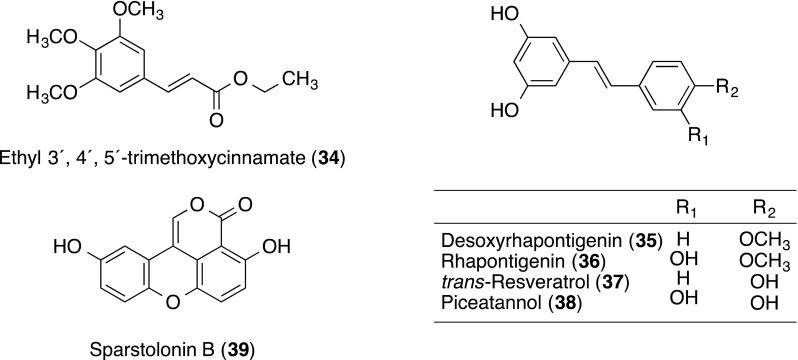


### Plant extract, etc.

Effects of crude plant extract, snake venom and other naturally occurring fatty acid derivatives on cell adhesion molecules are also reported. As they are not isolated as pure components, only references are shown [36–45].

### Cell adhesion inhibitors of microbial origin

Macrosphelides (MSs) are 16-membered macrolides, embodying 3 ester linkages produced by several fungal strains. MSs A–D (**40**–**43**), J (**49**) and K (**50**) were originally isolated from the culture broth of *Microsphaeropsis* sp. FO-5050 as cell adhesion inhibitors [46–49]. At almost the same time, MSs A (**40**), C (**42**), E–I (**44**–**48**), L (**51**), *seco*-MS E (**52**) and MS-M (**53**) were isolated from a fungal strain of *Periconia byssoides* originally separated from the sea hare *Aplysia kurodai* [50–54].

MSs A–D (**40**–**43**) inhibited the adhesion of SLe^x^-expressing HL-60 cells to endothelial cell leukocyte adhesion molecule 1 (ELAM-1)-expressing HUVECs in a dose-dependent fashion, with IC_50_ values of 3.5, 36, 67.5 and 25 µM, respectively. Among MSs, MS-A (**40**) showed the most potent inhibitory activity. On the one hand, MSs J (**49**) and K (**50**) were inactive (IC_50_ value: > 100 µg/mL).

It was suggested that they prevent the cell–cell adhesion by blocking the binding of SLe^x^ to ELAM-1. However, MSs showed no effect on the adhesion of sialyl Lewis A (SLe^a^)-expressing HL-60 cells to HUVECs. Furthermore, pretreatment of HL-60 cells, not HUVECs, with MSs caused inhibition of the adhesion of HL-60 to HUVECs. These findings indicated that MSs specifically bound to SLe^x^ on HL-60 cells to block the cell–cell adhesion [55].

MSs proved to be effective in several in vivo models. In the mouse model of B16/BL6 melanoma lung metastasis, MS-B (**41**) caused a dose-dependent decrease in lung metastatic nodules without any toxic effect including body weight loss in the range of 5–20 mg/kg. Furthermore, its efficacy in combination therapy with anti-cancer drugs was demonstrated. Combined therapy of MS-B (**41**) and cisplatin (CDDP) induced remarkable lung metastasis inhibition without adverse effects of CDDP to the host [56, 57].

The total synthesis of MS-A (**40**) has been reported by several groups. A novel total synthesis have been accomplished by the group of the Kitasato Institute [58, 59]. The combinatorial synthesis of a 122-member MS library including MSs A (**40**), C (**42**), E (**44**) and F (**45**) has been achieved based on a unique strategy for a three-component coupling utilizing a palladium-catalyzed chemoselective carbonylation and an unprecedented macrolactonization on a polymer support [60]. Synthetic approaches to MS derivatives, based on medicinal chemistry, were reviewed [61]. At present, MS-A (**40**) is commercially available as a reagent.


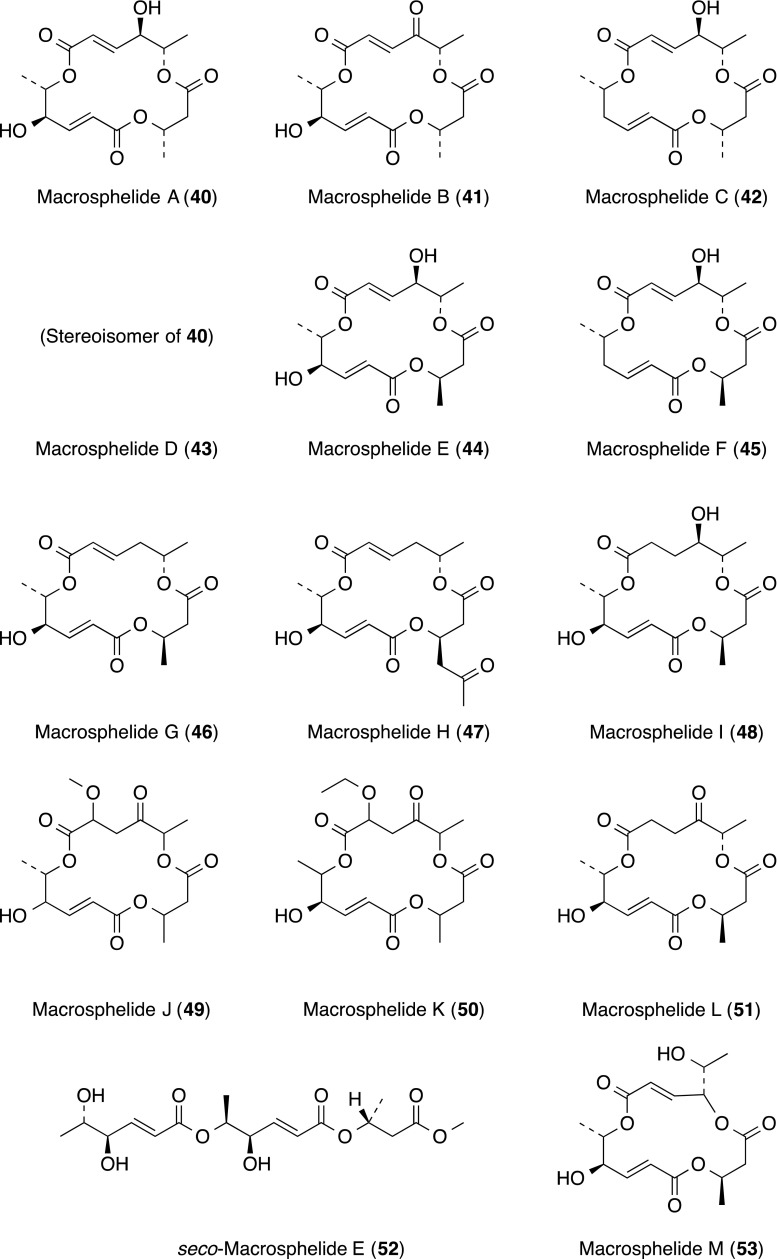


HUN-7293 (**54**) is a fungal cyclodepsipeptide that was first identified as an inhibitor of VCAM expression [62]. HUN-7293 (**54**) inhibited expression of VCAM-1 and ICAM-1 on TNF-α-stimulated human microvascular endothelial cells (HMEC-1), with IC_50_ values of 2 and 50 nM, respectively.

HUN-7293 (**54**) almost inhibited cell adhesion between the human Burkitt’s lymphoma B (BL 2) cell and TNF-α-stimulated HMEC-1 at a concentration of 20 nM. Total synthesis of **54** had done and evaluation of synthetic analogues as inhibitors of VCAM-1 expression was further reported [63, 64].

Nakagawa and co-workers found two inhibitors adxanthromycins A (**55**) and B (**56**) of ICAM-1/LFA-1-mediated cell adhesion molecule produced by *Streptomyces* sp. NA-148 [65–67]. The structure of **55** and **56** were characterized as dimeric anthrone peroxide skeleton containing an α-d-galactose for **55** and two α-d-galactose for **56**.

Both **55** and **56** inhibited homotypic aggregation of Epstein–Barr virus (EBV)-immortalised B cell lymphoblastoid line (JY cell) from 1.5 µg/mL in a dose dependent manner. A complete inhibition was observed at 6.25 µg/mL.

The toxicity (IC_50_) of **55** and **56** against JY cell was 15.2 µg/mL. Compounds **55** and **56** also inhibited SKW-3 adhesion to soluble ICAM-1 in a dose-dependent manner with an IC_50_ of 18.8 and 25.0 µg/mL, respectively. The cell toxicity (IC_50_) of adxanthromycins against SKW-3 was 110.0 µg/mL. In the cell-free receptor binding assay, both **55** and **56** showed weak inhibition with an IC_50_ of 760 µg/mL. They were reported as the first example of inhibitors of ICAM-l/LFA-1-mediated adhesion molecule isolated from microbial sources.

The benzopyran derivative (**57**) was found in the culture of *Streptomyces* sp. Mer-88 as ICAM-1/LFA-1 binding inhibitors, ICAM-1 inhibitors and LFA-1 inhibitors [68]. Compound **57** inhibited binding between ICAM-1 and LFA-1 in the range of 31–2500 µg/mL dose dependently, without cytotoxicity up to a concentration of 1000 µg/mL. Then, Xu and co-workers reported isolation of the same compound, *N*-[[3,4-dihydro-3*S*-hydroxy-2*S*-methyl-2-(4′*R*-methyl-3′*S*-pentenyl)-2*H*-1-benzopyran-6-yl]carbonyl]-threonine (**57**), produced by *Streptomyces xiamenensis*, its structure, including the absolute configuration, and its anti-fibrotic properties [69].


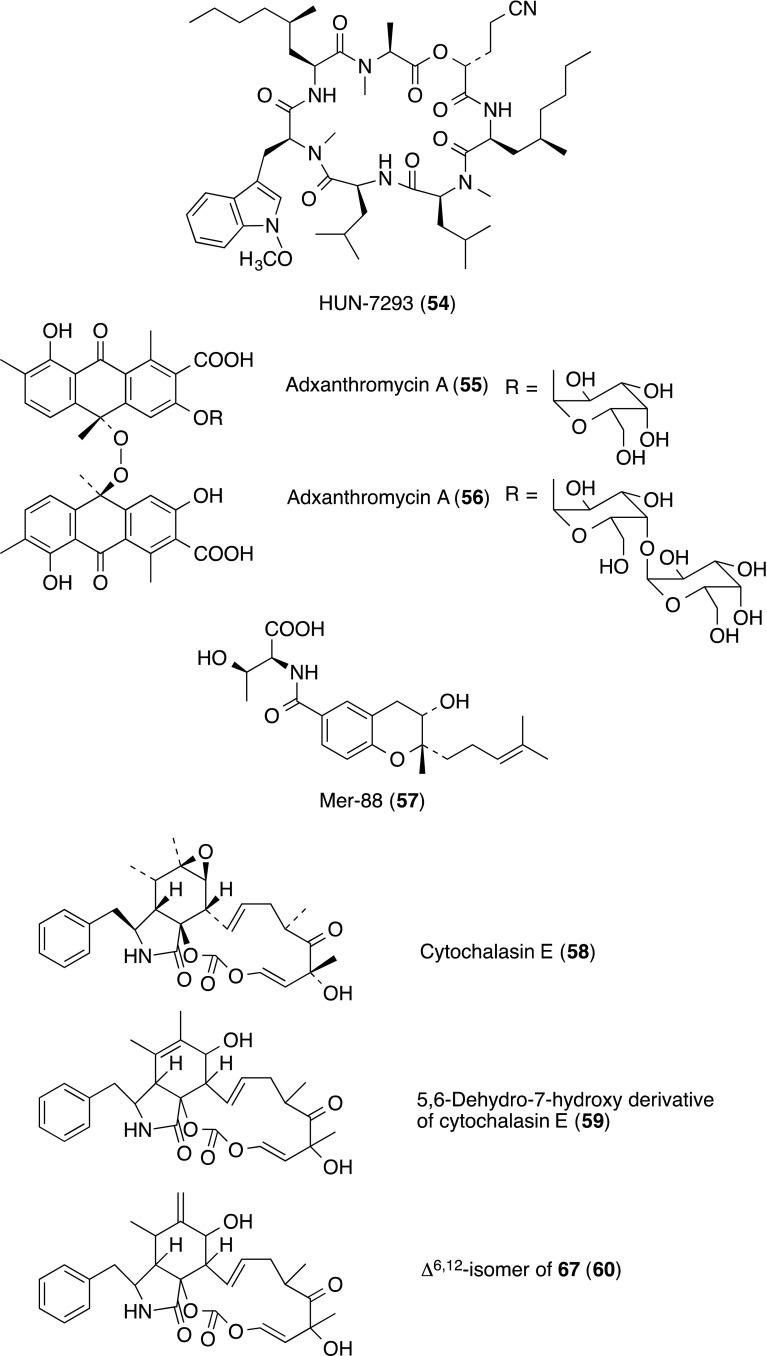


Three derivatives of cytochalasin were isolated from the cultured broth of the fungal strain *Mycotypha* sp. UMF-006, as inhibitors of cell adhesion based on LFA-1/ICAM-1 [70]. These compounds were identified to be cytochalasin E (**58**), 5,6-dehydro-7-hydroxy derivative of cytochalasin E (**59**) and Δ^6,12^-isomer of **59** (**60**). All these components inhibited adhesion of HL-60 cells to CHO-ICAM-1 cells at IC_50_ values of 30 µg/mL for **58**, 75 µg/mL for **59** and 90 µg/mL for **60**.

Members of the efomycine family from *Streptomyces* BS1261 were found to inhibit leukocyte adhesion, from a screening library of 20,000 natural compounds [71]. Finally, efomycines A B, E and G (**61**) were isolated as active substances [only structures of G (**61**) and M (**62**) were shown]. Members of the efomycine family inhibited the binding of human or porcine neutrophils by 50–60% at 10^−5^ M, whereas efomycine M (**62**) did not have a significant effect. Efomycine M (**62**) showed the most selective inhibitory effects on selectin-mediated leukocyte-endothelial adhesion in vitro, significantly diminishing rolling in mouse ear venules in vivo. In addition, efomycine M (**70**) alleviated cutaneous inflammation in two complementary mouse models of psoriasis, one of the most common chronic inflammatory skin disorders. Molecular modeling demonstrated a spatial conformation of efomycines mimicking naturally occurring selectin ligands.

Three compounds, NP25301 (**63**), NP25302 (deoxybohemamine **64**) and bohemamine (**65**), inhibitors of cell adhesion based on LFA-1/ICAM-1, were isolated from the cultured broth of the strain *Streptomyces* sp. UMA-044 [72]. Compounds **63**–**65** inhibited adhesion of HL-60 cells to CHO-ICAM-1 cells at IC_50_ values of 29.5 µg/ml for **63**, 24.3 µg/ml for **64** and 27.2 µg/ml for **65**.

Random screening of chemical libraries identified the 3-hydroxy-3-methylglutaryl coenzyme A (HMG-CoA) reductase inhibitor lovastatin (**66**), a drug clinically used for lowering cholesterol levels, as an inhibitor of the LFA-1/ICAM-1 interaction [73]. Lovastatin (**66**) showed binding inhibition of recombinant ICAM-1 to purified LFA-1 with an IC_50_ value of 2.1 µM. Inhibitory effects of statin-derived compounds on the binding were also shown with a range of IC_50_ 0.04–14 µM. The biological relevance of LFA-1 inhibition by statins with respect to the overall benefit of this drug class was reviewed [74].

Structurally related to known naturally occurring cyclic heptadepsipeptides, HUN-7293 (**54**), named heptadepsin (**67**), was isolated from the culture broth of *Paenibacillus* sp. [75]. Compound **67** inhibited LPS-stimulated adhesion between HUVECs and HL-60 cells with an IC_50_ value of 0.92 µg/mL, without showing any cytotoxicity up to 30 µg/mL. Compound **67** also inhibited cellular adhesion induced by lipid A, the active component of LPS, but it did not inhibit TNF-α- or IL-1β-induced cell adhesion. Heptadepsin (**67**) was shown to inactivate LPS by direct interaction with LPS and lipid A from the results of surface plasmon resonance analysis.


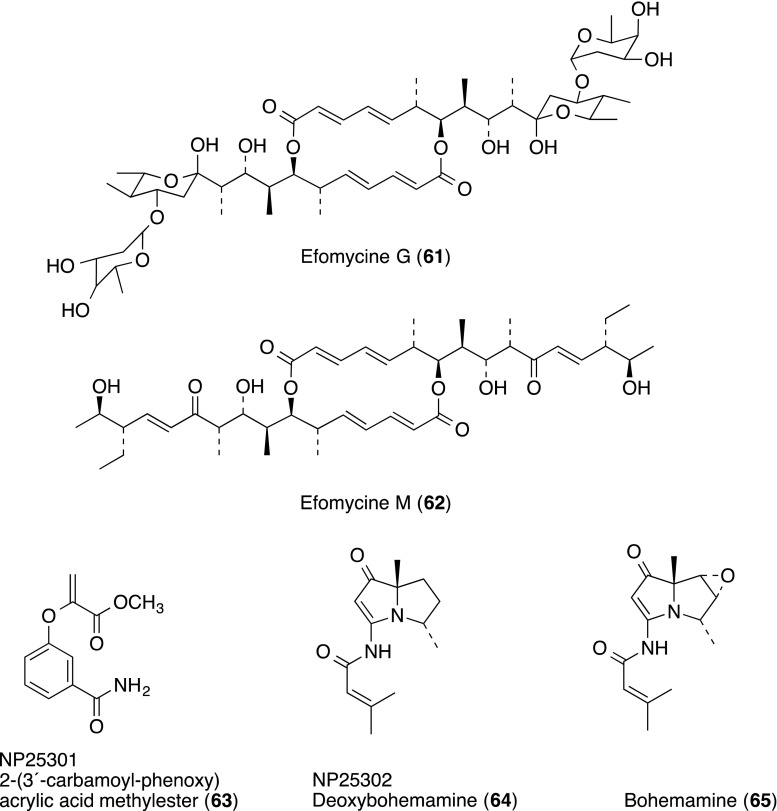



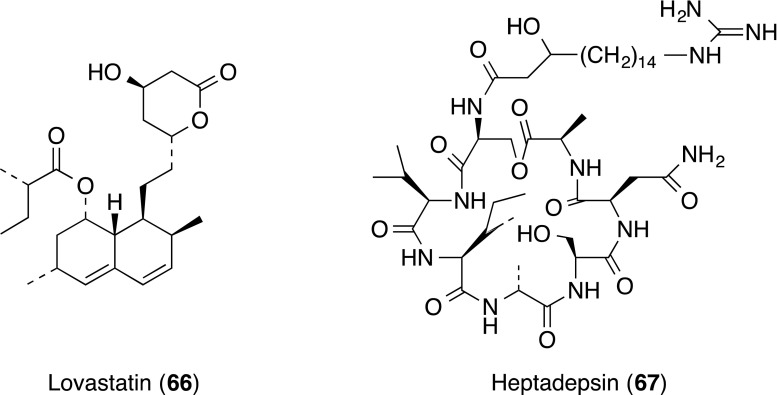


Peribysins A–J (**68**–**77**) including a furanofuran, were isolated from the culture of a strain *Periconia byssoides* OUPS-N133 separated from the sea hare *Aplysia kurodai*. They inhibited the adhesion of HL-60 cells to LPS-stimulated HUVECs with an IC_50_ range of 0.1–20 µM. Among them, compounds A (**68**) and D (**71**) showed the most potent cell adhesion inhibitory activity with IC_50_ values 0.3 and 0.1 µM, respectively, as compared to that of herbimycin A (standard, IC_50_ 38 µM) [54, 76–78]. Interestingly, the producing strain OUPS-N133 of peribysins was the same as that of MSs A (**40**), C (**42**), E (**44**), F–I (**45**–**48**), L (**51**) and M (**53**). The total synthesis necessitated revision of the assignment of the absolute configuration of **72** [79].


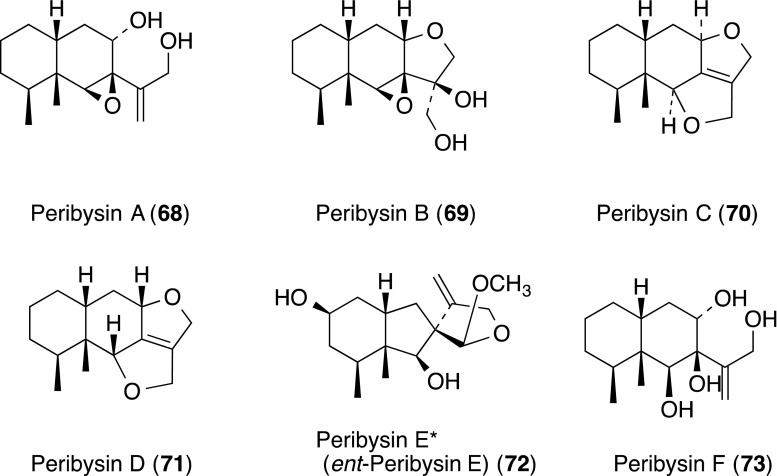



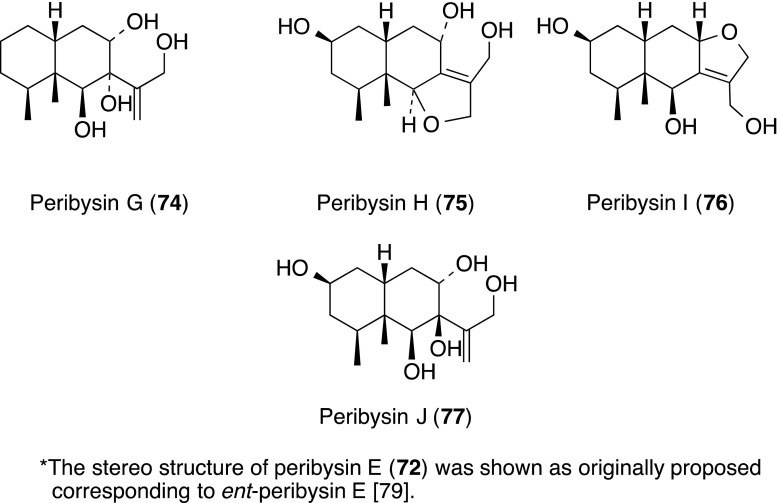


### Cell adhesion inhibitors derived from a marine organism

In the screening for P-selectin inhibitors, sulfonoquinovosyl dipalmitoyl glyceride (**78**) and phosphatidylglycerol (**79**) were isolated from the 85% EtOH extract of the marine alga *Dictyochloris fragrans*. Both **78** and **79** inhibited P-selectin binding to sulfatides in the P-selectin–IgG ELISA assay, with IC_50_ values of 5 and 1 µM, respectively [80].

The inhibitory effect of **78** on HL-60 cell adhesion to immobilized P-selectin receptor globulin (Rg) was only shown with an IC_50_ value of 40 µM. Compound **78** was further shown for its ability to inhibit (24%) the P-selectin-dependent binding of activated platelets to HL-60 cells.


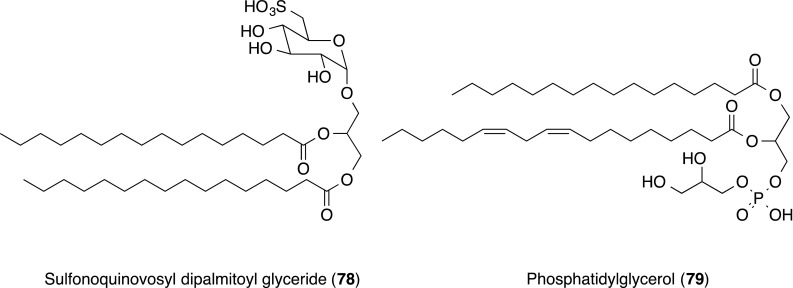


From a panel of 60 unusual marine natural products, 17 compounds inhibited LFA-1/ICAM-1-based cell aggregation without showing significant cytotoxicity in the primary assay. Six compounds inhibited the cell–cell adhesion of HL-60 cells to CHO-ICAM-1 cells. The unusual oxylipin *Cymathere* aldehyde methyl ester (**80**; IC_50_ 3.5 µM), cyanobacterial lipopeptide microcolins B (**81**; IC_50_ 0.15 µM) and D (**82**; IC_50_ 0.9 µM), bromophenol avrainvilleol (**83**; IC_50_ 2.2 µM), sesquiterpene cymopol (**84**; IC_50_ 2.7 µM) and cryptophyte-derived compound styrylchromone hormothamnione diacetate (**85**; IC_50_ 1.5 µM) significantly inhibited LFA-1/ICAM-1-mediated cell adhesion (Table 2). The pharmacological activity and structure–activity relationships of selected marine algal metabolites are described [81].

**Table 2:** Effects of marine natural products (**80**–**85**) on cell adhesion of HL-60 cells to CHO-ICAM-1 cells, and on proliferation of CHO-ICAM-1 cells

	Compound	Sources	HL-60			HL-60/CHO-ICAM-1	CHO-ICAM-1
			(A) C. A.^a^	(B) C. P.^b^	S.I.^c^	(C) Cell adhesion	(D) C. P.^b^
			MIC^d^ (µM)	IC_50_ (µM)	B/A	IC_50_ (µM)	IC_50_ (µM)
**80**	*Cymathere* aldehyde methyl ester	*Cymathere triplicate*	5.9	> 5.9	> 1.0	3.5 ± 0.3	2.7 ± 0.5
**81**	Microcolin B	*Lyngbya majuscula*	1.7	1.3 ± 0.2	0.7	0.15 ± 0.09	> 1.4
**82**	Microcolin D	*Lyngbya majuscula*	1.8	> 1.8	> 1.0	0.9 ± 0.1	1.1 ± 0.2
**83**	Avrainvilleol	*Avrainvillea longicaulis*	3.1	> 3.1	> 1.0	2.2 ± 0.2	0.8 ± 0.3
**84**	Cymopol	*Cymopolia barbata*	1.3	> 3.8	> 3.0	2.7 ± 0.2	> 3.1
**85**	Hormothamnione diacetate	*Chrysophaeum taylori*	2.6	> 2.6	> 1.0	1.5 ± 0.2	0.6 ± 0.2
	Cytochalasin B^e^		0.3	> 2.6	8.9	1.2 ± 0.2	0.2 ± 0.01

Values are means of three independent determinations ± SE

^a^Cell aggregation; ^b^Cell proliferation; ^c^Specific index; ^d^Minimum inhibitory concentration; ^e^Used as a standard


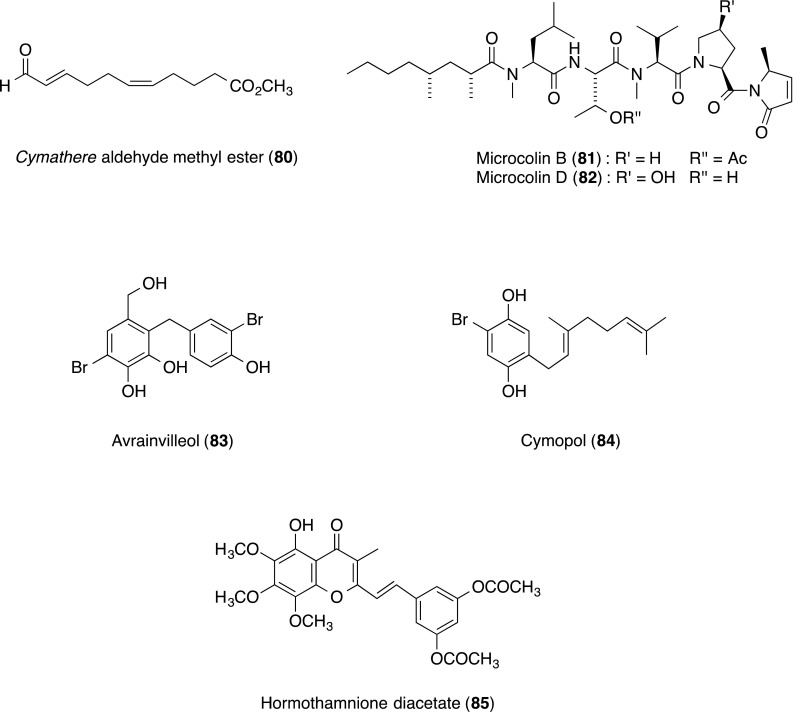


## Conclusions

As listed above, natural cell adhesion inhibitors are still proved essential to drug development due to their great variety of unexpected structures, such as plant-derived terpenoid (**1**–**24**), lignans (**25**–**26**), flavonoid (**27**) and alkaloid (**28**–**33**), and macrolide (**40**–**53**, **58**–**60**), cyclodepsipeptide (**54**, **67**), dimeric anthrone (**55**, **56**), and furanofuran (**68**–**77**) of microbial origin, and marine-derived compounds (**78**–**85**).

Among those from different origin, it is interesting to note that casearinols (**5**, **6**), casearinones (**7**, **8**), andrographolide (**9**), lovastatin (**66**) and peribysins A–J (**68**–**77**) were structurally similar based on a decalin (decahydronaphthalene) skeleton with a side chain or condensed furanofuran.

Basically, all inhibitors covered this time were found using a cell-based assay for cell–cell adhesion or cell-soluble cell adhesion molecule. In a different way from the cell-based assay, the pharmacophore of sLe^x^, recognized by a family of selectin, was used to search a three-dimensional database of chemical structures. As result of a search for a binding inhibitor between selectins and sLe^x^, glycyrrhizin (structure was not shown), a saponin and sweet-tasting constituent of *Glycyrrhiza glabra* (liquorice, Fabaceae) root, was matched as a pharmacophore [82].

Commercially available cell adhesion inhibitors are almost all developed based on peptide [83, 84]. But naturally occurring inhibitors such as MSs A–D (**40**–**43**) were not only first isolated in the culture of *Microsphaeropsis* sp. FO-5050 as cell adhesion inhibitors, but also possess a novel macrocyclic skeleton with tri-ester groups. Furthermore, MSs (**40**–**53**), nontoxic against HL-60 cells and HUVECs, have recently become an active area of research for anticancer drugs [61]. It is of great interest that MSs (**40**–**53**) were found in the culture of *Periconia byssoides* OUPS-N133, a strain producing peribysins A–J (**68**–**77**) [54, 76–78]. Application of a diversity of microorganisms displays an infinite number of possibilities and unexploited production capacity. Therefore, natural products are still of particular interest as seeds for drug discovery.
