# Correction to: Naturally occurring cell adhesion inhibitors

**DOI:** 10.1007/s11418-018-1234-6

**Published:** 2018-08-03

**Authors:** Satoshi Takamatsu

**Affiliations:** Division of Natural Medicine and Therapeutics, Department of Clinical Pharmacy, School of Pharmacy, Showa University, 1-5-8 Hatanodai, Shinagawa-ku, Tokyo, 142-8555 Japan

## Correction to: Journal of Natural Medicines 10.1007/s11418-018-1220-z

The article Naturally occurring cell adhesion inhibitors, written by Satoshi Takamatsu, was originally published electronically on the publisher’s internet portal (currently SpringerLink) on 19 May 2018 without open access. With the author(s)’ decision to opt for Open Choice the copyright of the article changed on 2 August 2018 to © The Author(s) 2018 and the article is forthwith distributed under the terms of the Creative Commons Attribution 4.0 International License (http://creativecommons.org/licenses/by/4.0/), which permits use, duplication, adaptation, distribution and reproduction in any medium or format, as long as you give appropriate credit to the original author(s) and the source, provide a link to the Creative Commons license and indicate if changes were made.

The original article was corrected.

